# Somatic mutations in renal cell carcinomas from Chinese patients revealed by whole exome sequencing

**DOI:** 10.1186/s12935-018-0661-5

**Published:** 2018-10-17

**Authors:** Jie Wang, Zhijun Xi, Jianzhong Xi, Hanshuo Zhang, Juan Li, Yuchao Xia, Yuanxue Yi

**Affiliations:** 1 Department of Urology, Peking University First Hospital and Institute of Urology, National Research Center for Genitourinary Oncology, No 8, Xishiku Street, Xicheng District, Beijing, China; 2 Department of Biomedical Engineering, College of Engineering, Peking University, No 5, Yiheyuan Road, Haidian District, Beijing, China; 3 Beijing Genex Health Technology Co., Ltd., Beijing, China; 4 Chongqing Institute of Innovation and Entrepreneurship for Precision Medicine, Chongqing, China

**Keywords:** Renal cell carcinoma, Whole exome sequencing, Somatic mutation, Gene, PD-L1

## Abstract

**Background:**

While the somatic mutation profiles of renal cell carcinoma (RCC) have been revealed by several studies worldwide, the overwhelming majority of those were not derived from Chinese patients. The landscape of somatic alterations in RCC from Chinese patients still needs to be elucidated to determine whether discrepancies exist between Chinese patients and sufferers from other countries and regions.

**Methods:**

We collected specimens from 26 Chinese patients with primary RCC, including 15 clear cell renal cell carcinoma (ccRCC) samples, 5 papillary renal cell carcinoma (PRCC) samples and 6 chromophobe renal cell carcinoma (ChRCC) samples. Genomic DNAs were isolated from paired tumor-normal tissues and subjected to whole exome sequencing (WES). Immunohistochemistry analysis was performed to detect the programmed death ligand 1 (PD-L1) expression in tumor tissues.

**Results:**

A total of 1920 nonsynonymous somatic variants in exons and 86 mutations at splice junctions were revealed. The tumor mutation burden of ccRCC was significantly higher than that of ChRCC (P < 0.05). For both ccRCC and PRCC, the most frequent substitution in somatic missense mutations was T:A > A:T, which was different from that recorded in the COSMIC database. Among eight significantly mutated genes in ccRCC in the TCGA database, six genes were verified in our study including *VHL* (67%)*, BAP1* (13%)*, SETD2* (13%), *PBRM1* (7%)*, PTEN* (7%) and *MTOR* (7%). All the mutations detected in those genes had not been reported in ccRCC before, except for alterations in *VHL* and *PBRM1*. Regarding the frequently mutated genes in PRCC in our study, *DEPDC4* (p.E293A, p.T279A), *PNLIP* (p.N401Y, p.F342L) and *SARDH* (p.H554Q, p.M1T) were newly detected gene mutations predicted to be deleterious. As the most recurrently mutated gene in ChRCC in the TCGA dataset, *TP53* (p.R81Q) was somatically altered only in one ChRCC case in this study. The HIF-1 signaling pathway was the most affected pathway in ccRCC, while the PI3K-Akt signaling pathway was altered in all of the three RCC types. Membranous PD-L1 expression was positive in tumor cells from 6/26 (23%) RCC specimens. The PD-L1-positive rate was higher in RCC samples with the somatically mutated genes *CSPG4*, *DNAH11*, *INADL* and *TMPRSS13* than in specimens without those (P < 0.05).

**Conclusions:**

Using WES, we identified somatic mutations in 26 Chinese patients with RCC, which enriched the racial diversity of the somatic mutation profiles of RCC subjects, and revealed a few discrepancies in molecular characterizations between our study and published datasets. We also identified numerous newly detected somatic mutations, which further supplements the somatic mutation landscape of RCC. Moreover, 4 somatically mutated genes, including *CSPG4*, *DNAH11*, *INADL* and *TMPRSS13*, might be promising predictive factors of PD-L1-positive expression in RCC tumor cells.

**Electronic supplementary material:**

The online version of this article (10.1186/s12935-018-0661-5) contains supplementary material, which is available to authorized users.

## Background

Renal cell carcinoma (RCC) is one of the most common human malignancies, with an estimated 63,990 new cases and 14,400 deaths occurring annually in the United States [1]. In China, RCC is not reported among the top 10 cancer incidences and mortalities [2]. Among the different histological subtypes of RCC, clear cell renal cell carcinoma (ccRCC) is the most common type, followed by papillary renal cell carcinoma (PRCC) and chromophobe renal cell carcinoma (ChRCC). The molecular profiles of those three common subtypes of RCC have been studied using next generation sequencing (NGS) in a multitude of research projects such as The Cancer Genome Atlas (TCGA) and other projects from Japan, the European Union and France.

In ccRCC, *VHL* is the gene most frequently altered by germline and somatic mutations. According to TCGA analysis, *VHL, PBRM1, BAP1* and *SETD2* are the four most frequently somatically mutated genes in human ccRCC, all of which are typically mutated in combination with the loss of chromosome 3p, followed by *KDM5C, PTEN, MTOR* and *TP53* [3]. PRCC consists of two subtypes, type 1 and 2, based on distinct histological and genetic characteristics. In the TCGA database, several significantly mutated genes have been identified, including *MET, SETD2, NF2, KDM6A, SMARCB1, FAT1, BAP1, PBRM1, STAG2, NFE2L2* and *TP53*. Notably, somatic mutations in *MET* are mainly found in type 1 PRCC, whereas type 2 PRCC is primarily associated with somatic mutations in *SETD2, BAP1* and *PBRM1*, all of which are also frequently mutated in human ccRCC. Furthermore, *TFE3* and *TFEB* gene fusion and loss of *CNKD2A* have been shown to be dominant in type 2 PRCC [4]. In contrast to ccRCC and PRCC, ChRCC mainly manifests copy number variations of chromosomes, while relatively few somatic mutations are shown. *TP53* is the most recurrently somatically mutated gene in the TCGA dataset, followed by *PTEN* [5].

Up to now, the overwhelming majority of genomic datas of RCC have originated from the USA and European countries. As a consequence, most specimens have been collected from Caucasian and black patients, while very few Asian patients have been included. In the cBioPortal for Cancer Genomics (http://www.cbioportal.org), only 98 ccRCC samples from Japanese patients have been investigated. According to the International Cancer Genome Consortium (ICGC) Data Portal (https://dcc.icgc.org), only 10 Chinese donors are available in kidney cancer projects. The discrepancy between the somatic mutation profiles of RCC from Chinese patients and the published data still requires elucidation.

As a biomarker of response to the immune checkpoint inhibitor, PD-L1 expression in tumor cells was shown to correlate with the efficacy of immunotherapy involving programmed death 1 (PD-1)/PD-L1 inhibitors in many cancers. A recent study indicated that a longer progression-free survival was achieved with nivolumab plus ipilimumab than with sunitinib among advanced RCC patients with ≥ 1% PD-L1 expression but not among those with < 1% PD-L1 expression. Furthermore, PD-L1 was shown to serve as a predictive factor in terms of response and overall survival benefit from the nivolumab plus ipilimumab combination or nivolumab monotherapy as second-line treatment [6]. However, the association between PD-L1 expression and somatic mutations in RCC has not been widely investigated.

In this study, we aimed to uncover the somatic alterations in RCC from Chinese patients diagnosed with primary RCC including ccRCC, PRCC and ChRCC by using WES, as well as tried to find some correlations between somatic mutations and PD-L1 expression.

## Methods

### Patients and samples

Cancerous and paracancerous tissues were collected from patients with RCC who underwent either radical nephrectomy or partial nephrectomy at the Department of Urology of Peking University First Hospital. These tissues were promptly frozen in liquid nitrogen during the surgery and then stored at − 80 °C in our departmental tissue bank. A total of 26 RCC specimens with paired tumor-normal freshly frozen tissues were included in the present study, including 15 ccRCC specimens, 5 PRCC specimens and 6 ChRCC specimens. The pathological characteristics of these specimens were confirmed by pathologists. The study was approved by the Biomedical Research Ethics Committee of Peking University First Hospital, and written informed content was acquired from all enrolled patients.

### DNA extraction and WES

Genomic DNA (gDNA) was extracted from those tissues using TIANamp Genomic DNA Kit (Tiangen, China) according to the manufacturer’s instructions. The quality and quantity of the DNA were evaluated using the Qubit 3 Fluorometer (Invitrogen, United States), the Agilent 2100 Bioanalyzer (Agilent, United States) and agarose gel electrophoresis. The library was prepared using NEBNext DNA Library Prep Master Mix Set for Illumina (New England BioLabs, United States). Briefly: 200 ng of gDNA from each specimen was fragmented. The barcoded fragments were purified by XP beads and hybridized to the “capture library” containing specially designed probes. Subsequently, the hybridized DNA fragments were captured using streptavidin-coated beads, and the captured libraries were amplified with indexing primers and then purified. The quantity and quality of the final library were evaluated by the Qubit 3 Fluorometer and Agilent 2100 Bioanalyzer respectively. Meanwhile, qPCR was used to quantify each index-tagged library. At last, sequencing was performed on the Illumina Hiseq 2000 platform. The tumor tissue sequencing depth was set to 200×, and the paracancerous tissue sequencing depth was set to 100×.

### Data analysis

The short reads were first aligned to the hg19 reference genome using the Burrows Wheeler Aligner (BWA). The alignments were then recalibrated and filtered by the Genome Analysis Toolkit (GATK) [7]. MuTect2 was then applied to identify somatic mutations by comparing tumors against paracancerous tissues. Somatic variants were further filtered if the sequencing depth was below 10×, the coverage was below 5 reads or the mutation frequency was below 1%.

All somatic variants were annotated by Annovar [8]. The functional impacts of missense mutations were predicted by SIFT, PolyPhen2 HDIV, PolyPhen2 HVAR, LRT, MutationTaster, MutationAssessor, and FATHMM. The variants were considered deleterious mutations if they were scored by at least two algorithms as deleterious. Missense mutations that were not scored by those algorithms were classified as “unavailable” and excluded from the analysis. Other variants, including nonsense, frameshift and canonical ± 1 or ± 2 splice site mutations, were deemed pathogenic. This classification is consistent with the standards and guidelines of the American College of Medical Genetics (ACMG) [9].

The lollipop plot and oncoprint diagram were created using the Mutation Mapper and Oncoprint tools respectively [10, 11]. The tumor mutation burden (TMB), an emerging biomarker of immunotherapy responses, was calculated for each case [12]. The major signaling pathways associated with RCC in which genes were somatically mutated were analysed using the Kyoto Encyclopedia of Genes and Genomes (KEGG) database (http://www.genome.jp/kegg/pathway.html) [13].

### Immunohistochemistry and PD-L1 quantification

After all haematoxylin and eosin (H&E) tumor slides were reviewed by two pathologists, the corresponding formalin-fixed and paraffin-embedded blocks from the 26 RCC specimens were prepared into slides. All tumor slides were de-paraffinized and stained for PD-L1 using standard IHC techniques. The optimal dilution of the PD-L1 Rabbit mAb (E1L3 N; Cell Signaling Technology, Danvers, Massachusetts) was 1:200. All stained slides were assessed by two pathologists who were blinded to the clinical outcomes. The PD-L1 immunoreactivity in tumor cells was scored as follows: strong positive (++ to +++), > 5% stained cells with moderate or strong staining; weakly positive (+), 1–5% stained cells with any intensity; negative (−), < 1% stained cells.

### Statistical analysis

Correlations between the histological subtypes of RCC and the TMB were evaluated by the Mann–Whitney U test, and associations between PD-L1 expression and somatically altered genes were analysed via the Fisher’s exact test. *P *< 0.05 was considered statistically significant. SPSS 23.0 (USA) was employed to perform all the tests.

## Results

### Clinical and pathological characteristics of patients

In this study, 26 RCC cases consisted of 15 ccRCC cases, 6 ChRCC cases and 5 PRCC cases. In total, 9 females and 17 males were included. The median age was 59. All the patients suffered from primary RCC and none manifested distant or lymphatic metastasis. Details of clinical and pathological characteristics of the 26 patients with RCC are listed in Table 1.

**Table 1:** clinical and pathological information of RCC patients

Sample ID	Gender	Age at Diagnosis	Surgical Approach	Histological Subtype	Laterality	Tumor Grade	TNM Stage	Tumor Stage
1	M	44	LSRN	ccRCC	Left	G3	T3aN0M0	III
3	M	33	LSPN	ccRCC	Left	G1	T1aN0M0	I
4	M	60	LSRN	ccRCC	Left	G2	T1aN0M0	I
5	F	52	LSPN	ccRCC	Left	G1	T1aN0M0	I
6	M	59	LSPN	ccRCC	Left	G1	T1aN0M0	I
7	F	59	LSRN	ChRCC	Right	NA	T1bN0M0	I
8	F	70	LSPN	ccRCC	Right	G1	T1aN0M0	I
9	F	58	LSRN	ccRCC	Right	G2	T3aN0M0	III
10	M	49	LSRN	ccRCC	Right	G2	T2aN0M0	II
11	M	70	LSRN	ccRCC	Right	G1	T1bN0M0	I
12	M	76	LSRN	ccRCC	Right	G3	T3bN0M0	III
13	M	46	LSPN	ccRCC	Left	G3	T1aN0M0	I
14	M	63	LSRN	ccRCC	Right	G2	T1bN0M0	I
15	M	52	LSRN	ccRCC	Left	G1	T1bN0M0	I
25	F	43	ORN	ChRCC	Left	NA	T2bN0M0	II
36	M	38	LSRN	ccRCC	Left	G3	T1aN0M0	I
38	M	60	LSRN	ccRCC	Left	G1	T1bN0M0	I
39	F	24	LSRN	ChRCC	Right	NA	T1bN0M0	I
78	M	54	LSRN	ChRCC	Left	NA	T3aN0M0	III
82	F	76	LSPN	PRCC (II)	Right	G3	T1aN0M0	I
98	F	63	LSPN	ChRCC	Left	NA	T1aN0M0	I
114	M	65	LSRN	ChRCC	Right	NA	T1bN0M0	I
129	M	52	LSRN	PRCC (II)	Right	G2	T3aN0M0	III
130	F	78	LSRN	PRCC (II)	Right	G2	T1bN0M0	I
131	M	72	LSPN	PRCC (II)	Right	G2	T1aN0M0	I
137	M	38	LSPN	PRCC (I)	Right	G2	T1aN0M0	I

M, male; F, female; LSRN, laparoscopic radical nephrectomy; LSPN, laparoscopic partial nephrectomy; ORN, open radical nephrectomy; NA, not available

### Summary of somatic mutations

**Fig. 1:**
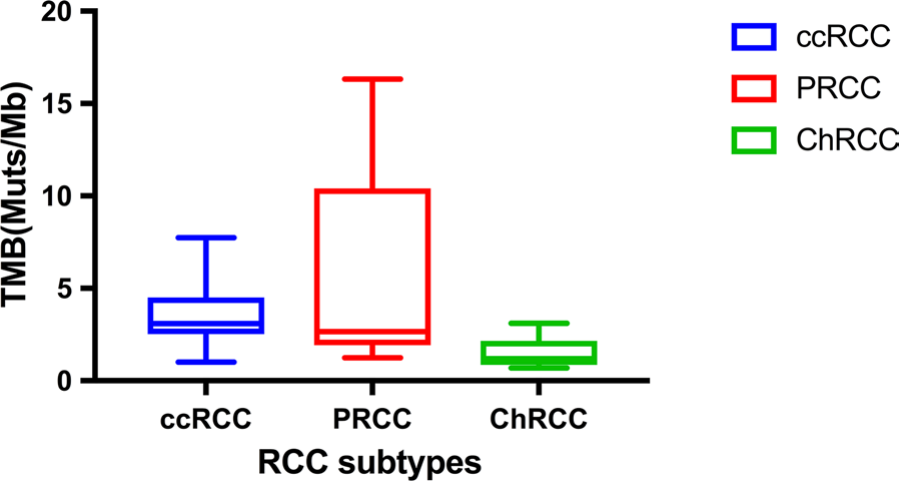
The box plot showing that the distribution of TMB in different RCC subtypes

In total, 1920 somatic nonsynonymous variants in exons and 86 mutations at splice junctions were revealed. Among all the somatic nonsynonymous variants, 1689 missense mutations, 139 stop-gain mutations, 84 frameshift mutations and 8 stop-loss mutations were identified. The TMB of ccRCC was significantly higher than that of ChRCC as revealed by the Mann–Whitney U test (P < 0.05), while the TMB of PRCC was not significantly different from that of ccRCC or ChRCC (P > 0.05) (Fig. 1). The TMB showed no statistical correlations with tumor grade, stage or size (P > 0.05).

**Fig. 2:**
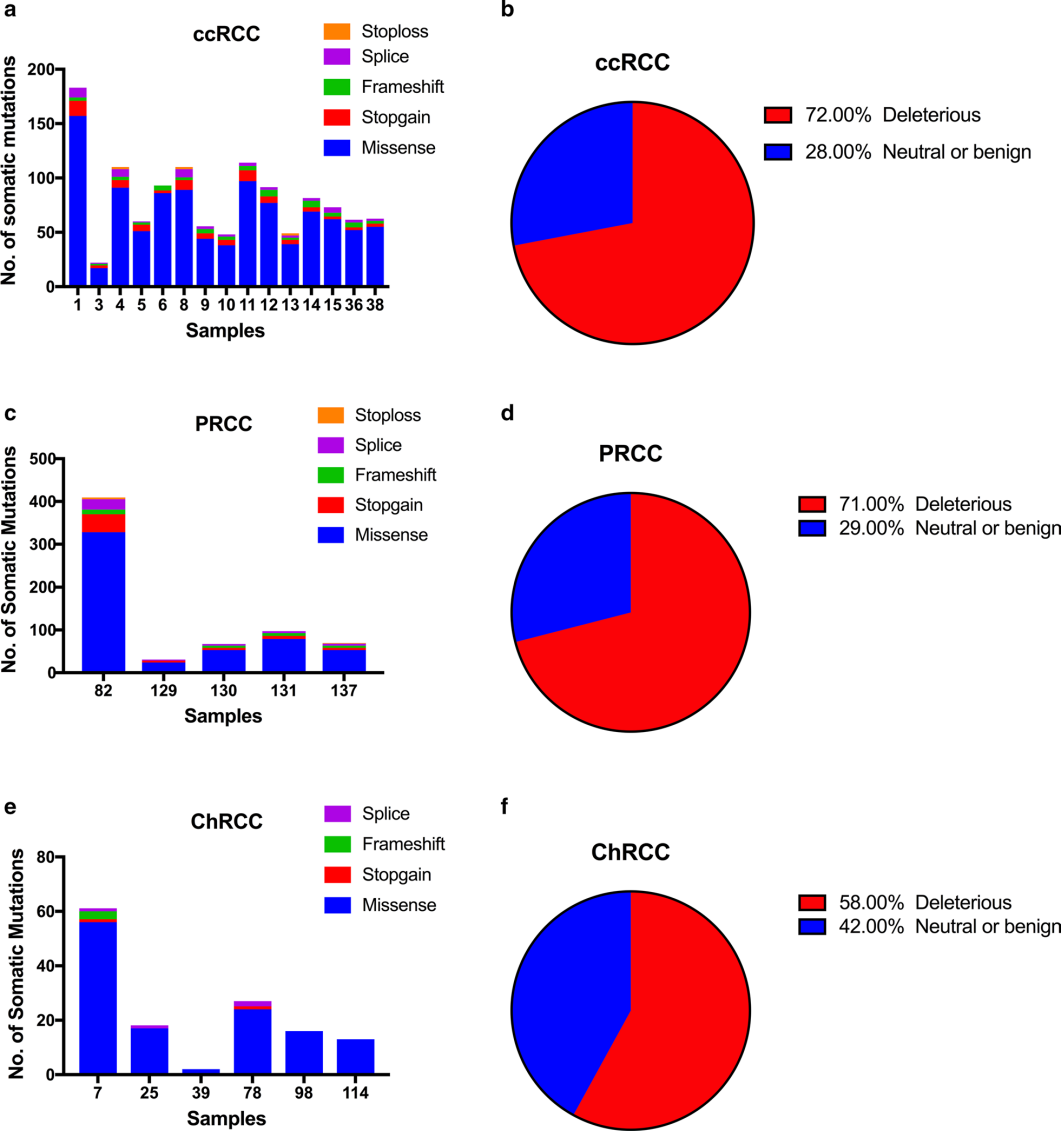
**a**, **c**, **e** Bar charts showing the number of somatic mutations identified in each patient based on different RCC subtypes. **b**, **d**, **f** Pie charts showing the frequency of functional impact of mutated genes according to protein prediction score

**Fig. 3:**
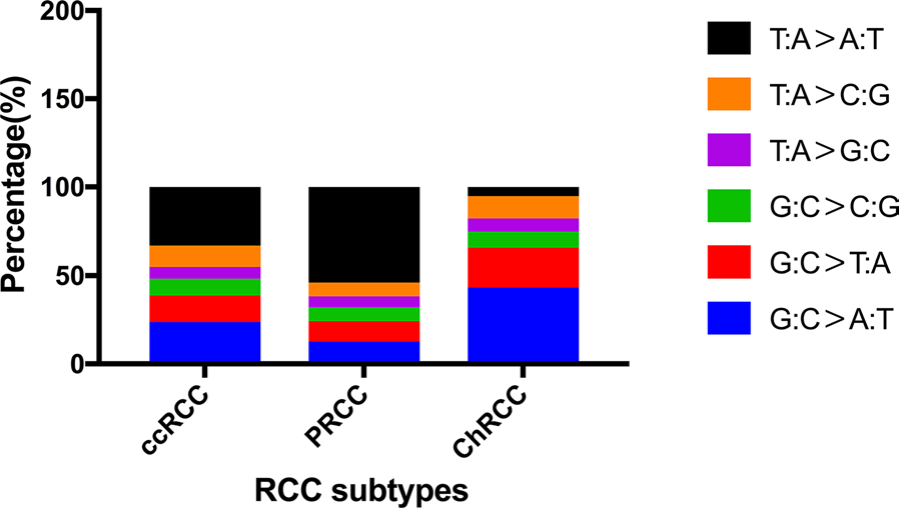
Bar charts showing that the percentage of different substitutions in missense mutations according to distinct RCC subtypes

**Fig. 4:**
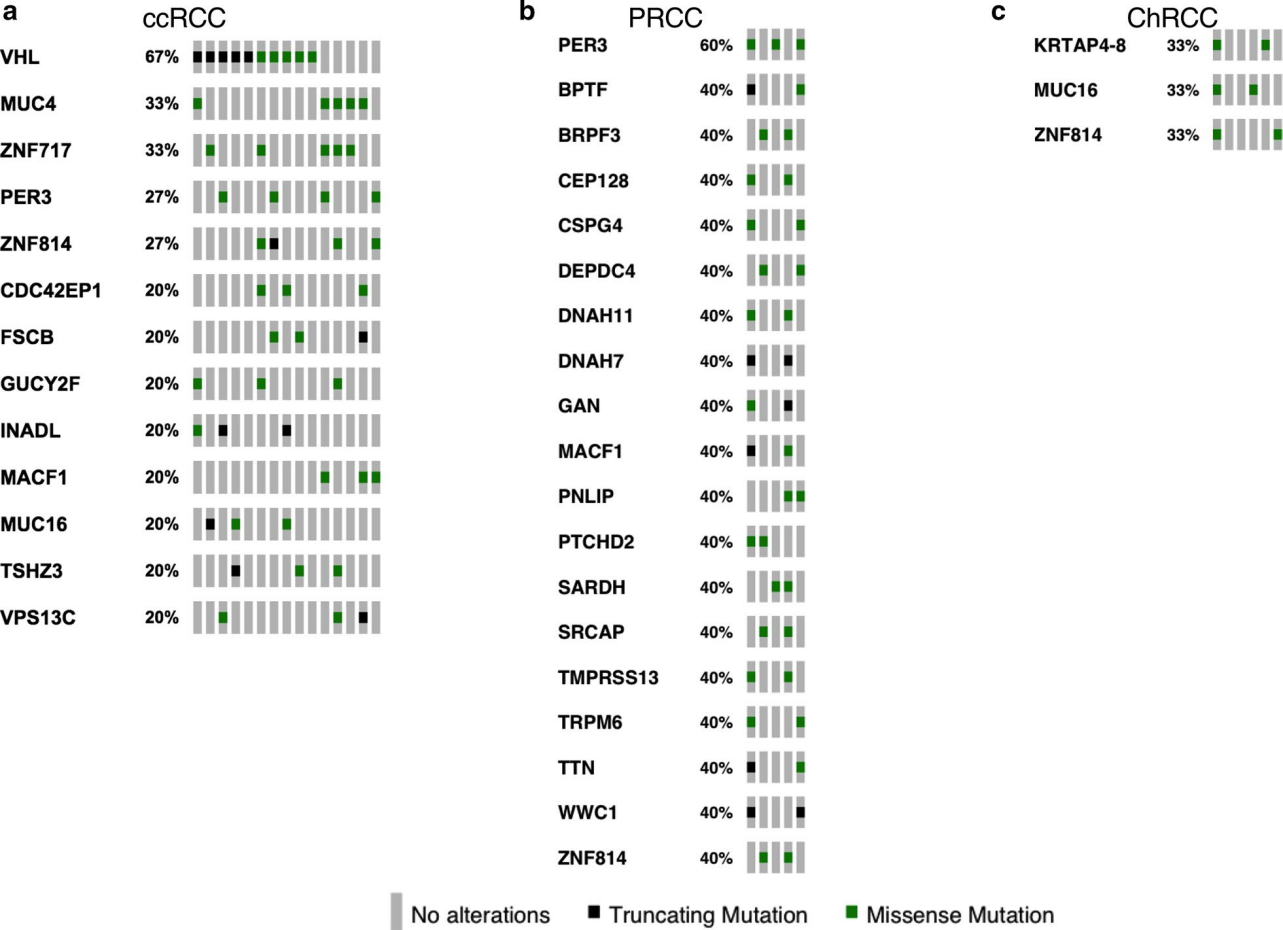
Oncoprint diagram **a** showing the mutated genes in at least three patients with ccRCC. Oncoprint diagram **b** and **c** illustrating the altered genes in at least two patients with PRCC and ChRCC respectively

**Fig. 5:**
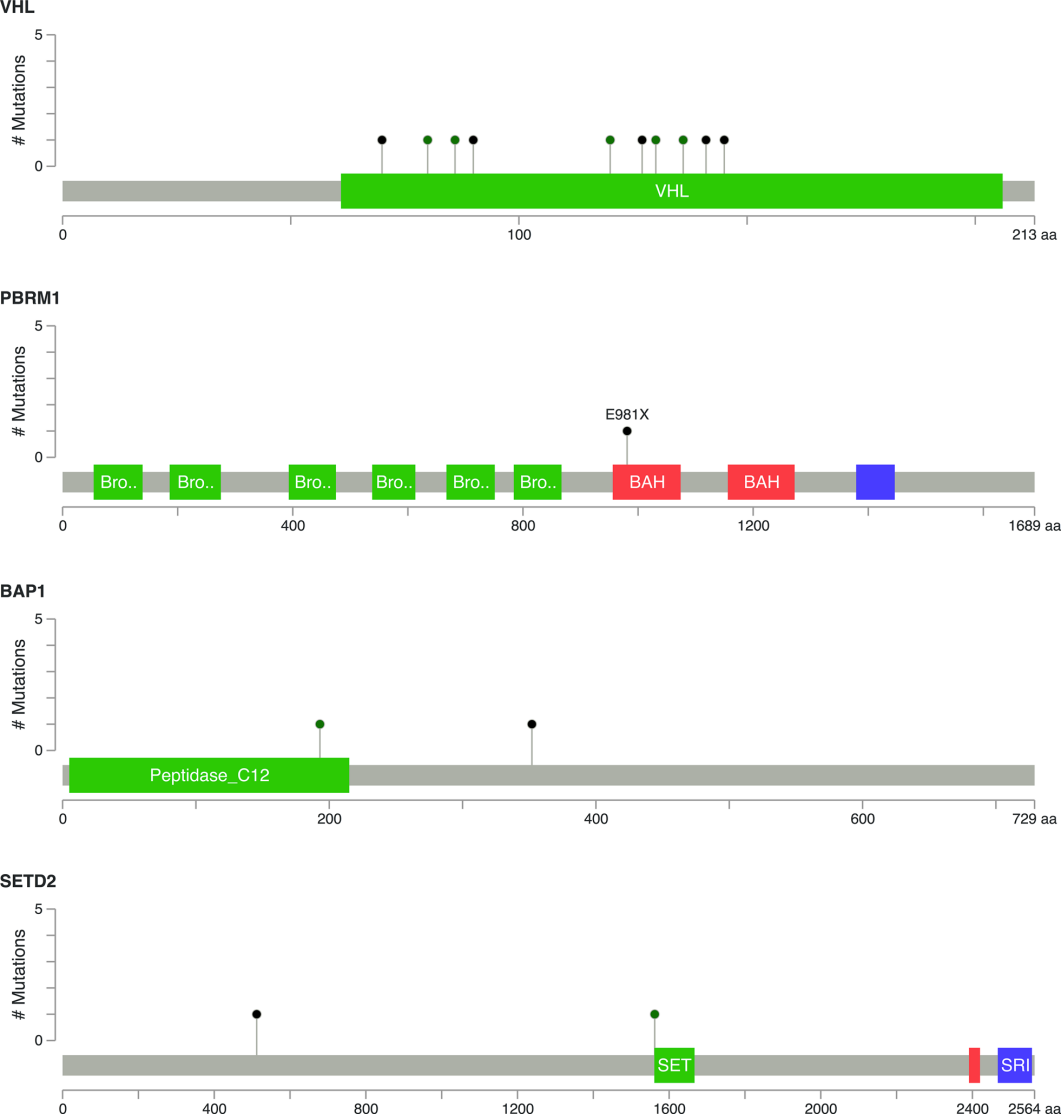
Mutation diagrams showing the distribution of somatic mutations on functional domains of VHL, PBRMl, BAPl and SETD2. The alterations were represented by circle and colors: green (missenses), black (truncating alterations)

In 15 ccRCC cases, we identified 1024 missense mutations, 81stop-gain mutations, 50 frameshift mutations, 48 splice mutations and 6 stop-loss mutations (Fig. 2a). Among all the missense mutations with available annotation information, 724 variants (72%) were predicted to be deleterious, and 277 mutations (28%) were predicted to be neutral or benign (Fig. 2b). The most frequent substitution in somatic missense mutations was exposed to be T:A > A:T, which was also the least common type in ChRCC cases (Fig. 3). In total, 13 mutated genes had a mutation frequency above 20%, each of which was altered in at least three samples (Fig. 4a). Consistent with previous studies, the most commonly mutated gene was *VHL* (10/15) in our study. These mutations contained five missense mutations (p.P86L, p.R120G, p.S80N, p.V130L, p.F136V), three frameshift deletions (p.G127fs, p.N141fs, p.N90fs) and two stop-gain mutations (p.E70X, p.Q145X). Those variants in *VHL* were located in the commonly known region of the VHL protein domain, all of which had been reported in the TCGA or COSMIC database (Fig. 5). Among the 12 most commonly mutated genes, only *CDC42EP1* had not been reported in ccRCC previously. In the *CDC42EP1* gene, the somatic missense mutation (S260P) was detected in three cases, which was not located in the protein domain for *CDC42EP1* and was predicted to be benign.

Regarding 5 PRCC cases, 537 missense mutations, 56 stop-gain mutations, 31 frameshift mutations, 34 splice mutations and 2 stop-loss mutations were detected (Fig. 2c). Among the 528 missense mutations with available annotation information, 375 variants (71%) were predicted to be deleterious and 153 mutations (29%) were forecasted to be neutral or benign (Fig. 2d). Like in the ccRCC cases, the most common substitution in missense mutations was T:A > A:T (Fig. 3). In total, 19 mutated genes were detected at a frequency above 40%, and each mutated gene was identified in at least two cases (Fig. 4b). *PER3* was the most commonly mutated gene observed in 3 PRCC cases (50%), which was also mutated in 4 ccRCC cases (27%). None of the variants detected in *PER3* were located in its protein domain and they were all predicted to be neutral or benign. Among the remaining frequently mutated genes, *DEPDC4* (p.E293A, p.T279A), *PNLIP* (p.N401Y, p.F342L) and *SARDH* (p.H554Q, p.M1T) had not been reported to correlate with PRCC before, and they were all predicted to be deleterious.

In the 6 ChRCC cases, 128 missense mutations, 2 stop-gain mutations, 3 frameshift mutations and 4 splice mutations were identified (Fig. 2e). Among the 124 missense mutations that had been annotated successfully, 72 variants (58%) were predicted to be deleterious, and 52 mutations (42%) were thought to be neutral or benign (Fig. 2f). The most recurrent substitution in missense mutations was G:C > A:T, which was distinct from that in ccRCC and PRCC cases (Fig. 3). Only 3 genes (*KRTAP4*–*8*, *MUC16*, *ZNF814*) were mutated at a frequency of 33%, and each gene mutation was uncovered in two cases (Fig. 4c). It’s worth noting that the *ZNF814* gene was also mutated in 4 ccRCC cases and 2 PRCC cases. Among all these mutations in *ZNF814* gene, p.P323H, p.R322K and p.G320E presented as a fixed combination occurring in three RCC types. Furthermore, p.P323H and p.G320E in *ZNF814* were predicted to be deleterious, while p.R322K was predicted to be benign. The *KRTAP4*–*8* gene had not been reported to be somatically altered in ChRCC previously. Among the 4 missense mutations in *KRTAP4*–*8*, p.V71M and p.S68R were forecasted to be deleterious, while p.H91R and p.K76R were predicted to be benign.

### Comparison with public databases

**Fig. 6:**
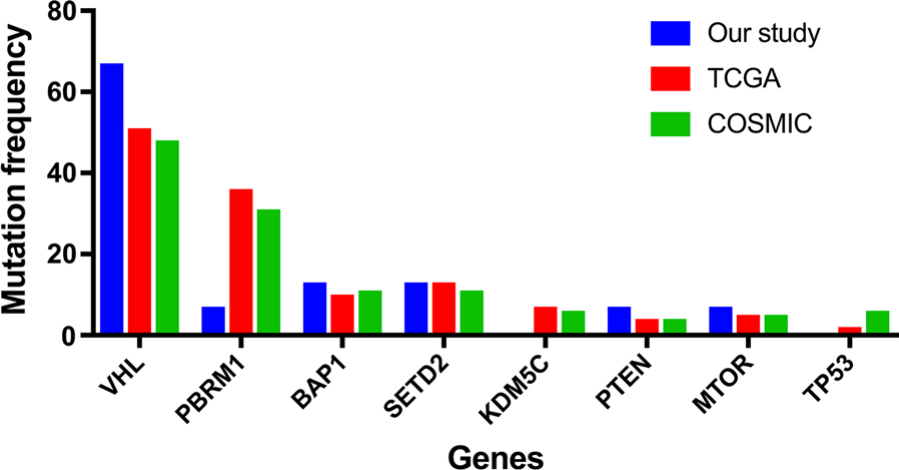
The bar chart showing that discrepancies of the mutation frequency of eight significantly mutated genes (*VHL*, *PBRMl*, *BAPl*, *SETD2*, *KDM5C*, *PTEN*, *MTOR*, *TP53*) between our study and two public datasets (TCGA and COSMIC)

In the COSMIC database, the most frequent substitution in missense mutations in ccRCC is G:C > A:T, which is different from what we found in this study (T:A > A:T). Among the top 8 frequently mutated genes (*VHL, PBRM1, BAP1, SETD2, KDM5C, PTEN, MTOR*, *TP53*) in ccRCC in the COSMIC database, which also represent the eight most significantly mutated genes in the TCGA database, six were verified in our study, including *VHL* (67%), *PBRM1* (7%), *BAP1* (13%), *SETD2* (13%), *PTEN* (7%) and *MTOR* (7%) (Fig. 6). It’s worth noting that the amino acid alterations p.P352fs and p.H193Q in *BAP1*, p.W1562C and p.S512X in *SETD2*, p.V343fs in *PTEN* and p.R882S in *MTOR* had not been reported previously in ccRCC, all of which were considered to be deleterious in this study. Figure 5 shows the distribution of somatic mutations identified in this study in functional domains for *VHL*, *PBRM1*, *BAP1* and *SETD2*.

For PRCC, the most recurrently mutated gene is *MET* in the COSMIC database (6%), which is also the most significantly mutated gene evaluated by MutSigCV in the TCGA database (7.45%). However, no mutations in *MET* gene was detected in our study. Notably, *PBRM1* gene that was mutated in one ccRCC case was also altered in one PRCC case (type 2), which was reported to be mutated at a frequency of 2% in the COSMIC database and 3.9% in the TCGA database. Moreover, in accordance with ccRCC, the most common substitution in missense mutations in PRCC in the COSMIC database is G:C > A:T, which is distinct from what we found in this study (T:A > A:T).

Both in the TCGA and COSMIC database, *TP53* is the most frequently mutated gene in ChRCC, with mutation frequencies of 30.77 and 11% respectively, which was also verified in one ChRCC case in this study. Moreover, the amino acid alteration p.R81Q in *TP53* had not been reported before and was predicted to be deleterious. In the COSMIC database, the most frequent substitution in missense mutations in ChRCC is G:C > A:T, which is consistent with our finding.

### Major signaling pathways altered in RCCs

The somatically mutated genes discovered in this study were used to evaluate the impact on the major signaling pathways associated with RCC, including the PI3K-Akt, mTOR, p53, HIF-1, Hippo, MAPK signaling pathways and the SWI/SNF complex [3, 4, 14]. According to our analysis, the HIF-1 signaling pathway (12/15) was the most affected pathway in ccRCC, in which *VHL* was the most frequently mutated gene (67%), followed by the PI3K-Akt signaling pathway (10/15). The PI3K-Akt signaling pathway (4/5) was the most influenced pathway in PRCC, followed by the Hippo signaling pathway (3/5) and the p53 signaling pathway (2/5). In ChRCC, a few mutated genes were identified as components of the signaling pathways mentioned above, including the PI3K-Akt (3/6), MAPK (2/6) and HIF-1 (2/6) signaling pathway. Notably, the *TP53* gene that was only mutated in one ChRCC case was involved both in the PI3K-Akt and MAPK signaling pathway, which was reported to be the most recurrently mutated gene in the TCGA database [5] (Additional file 1: Table S1).

### Association between PD-L1 expression and somatic mutations

**Fig. 7:**
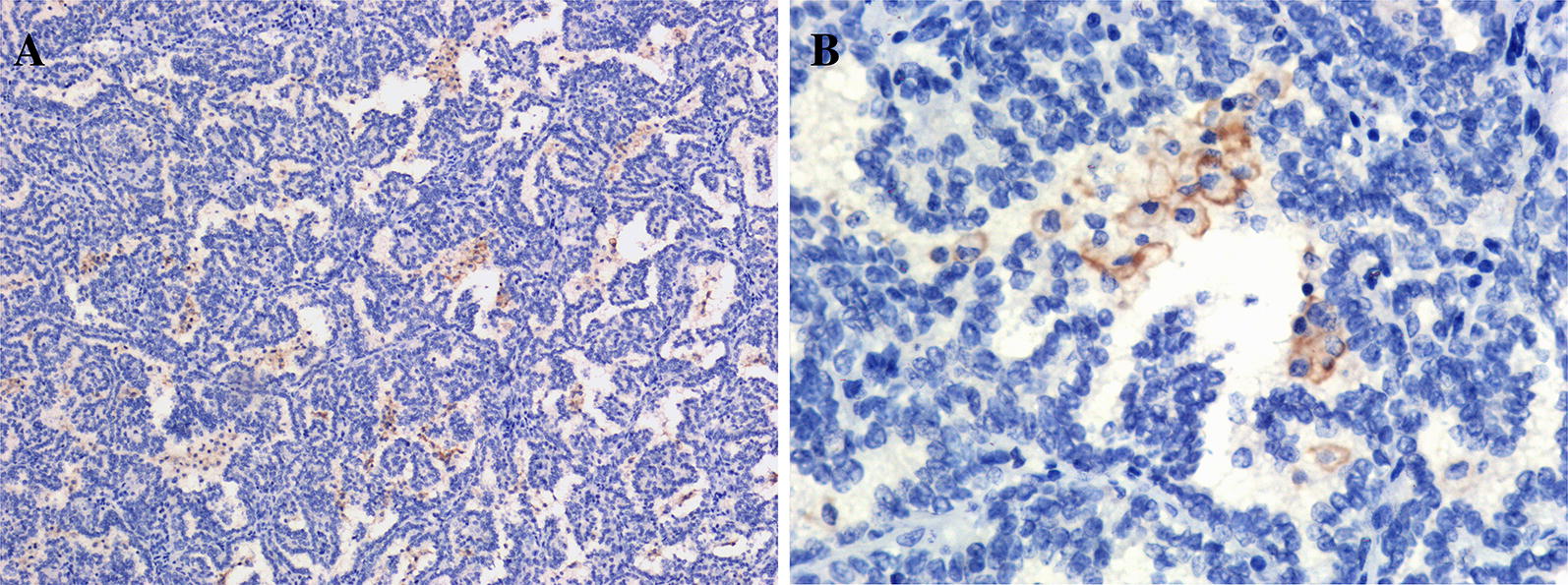
Immunohistochemical staining of PD-LI in RCC specimens . PD-LI membranous staining was identified in tumor cells. **A** ×10 magnification; **B** ×40 magnification

Membranous PD-L1 expression was positive in tumor cells from 6/26 (23%) RCC specimens, including 3 ccRCC samples, 2 PRCC samples and 1 ChRCC sample (Fig. 7). Only case 82 showed strong positivity (+++) in tumor cells for PD-L1 expression, in which the TMB (16.33 Muts/Mb) was the highest among 26 RCC cases, while the other 5 cases showed weak positivity (+). However, we didn’t find any statistical correlation between the TMB and PD-L1 expression (P > 0.05). In total, six genes were somatically mutated in two of the three PD-L1-positive ccRCC cases, including *VHL*, *INADL*, *MUC4*, *RAD21*, *CSPG4* and *BAP1*. Both of the two PD-L1-positive PRCC cases contained somatic alterations in six other genes, namely, *MACF1*, *DNAH7*, *DNAH11*, *TMPRSS13*, *CEP128* and *GAN*. In addition, *TMPRSS13* was also somatically mutated in one ccRCC case. Fisher’s exact test revealed that somatic mutations in *CSPG4*, *DNAH11*, *INADL* and *TMPRSS13* were significantly associated with PD-L1-positive expression in RCC tumor cells. Among the 26 RCC cases, the PD-L1-positive rate in tumor cells was higher in samples with the 4 somatically mutated genes, including *CSPG4*, *DNAH11*, *INADL* and *TMPRSS13*, than in samples without those (P > 0.05).

## Discussion

In recent years, the landscape of genomic somatic alterations in RCC has been depicted by several research projects including the TCGA, supported by multiple countries, which could be helpful for studying oncogenesis and new treatment strategies. However, racial differences might also contribute to the diversity of genomic somatic aberrations in tumors. For instance, in a study focusing on racial differences in the sequencing results of hereditary malignancies, Caswell and colleagues reported that a higher proportion of whites than nonwhites carried deleterious *CHEK2* mutations [15]. It’s well known that the vast majority of somatic mutation profiles associated with ccRCC, PRCC and ChRCC were unmasked by foreign researchers. The subjects were mainly Caucasian and black patients. Only 10 Chinese patients with ccRCC have been evaluated using WES before [16]. These data can be acquired via the ICGC data portal. The discrepancies and similarities of the somatic mutation landscapes in ccRCC, PRCC and ChRCC between Chinese patients and sufferers from other countries and regions still need to be elucidated with a larger sample size. In this study, we performed WES on paired fresh-frozen tissues obtained from 26 RCC cases.

In previous investigations, *VHL* was always reported to be the most frequently mutated gene in ccRCC. As revealed in a study about WES performed on 106 ccRCC specimens from Tokyo [14], *VHL* gene was somatically mutated at a frequency of 40.57%. According to the TCGA database [3], the mutation frequency of *VHL* was 51.42%, which was much lower (20%) in the WES study performed on 10 Chinese patients with ccRCC [16]. In our study on 15 paired tumor-normal ccRCC samples from Chinese patients, the mutation frequency of *VHL* was 66.67%, which was much higher than that in the WES study performed on 10 Chinese with ccRCC previously. The big difference in the mutation frequency of *VHL* between those two Chinese studies is probably due to the distinct sample amount, diverse sequencing platforms and different sample source centers. In this study, all of the somatic mutations in *VHL* were located in the known domain for *VHL* and determined to be deleterious to protein function. In other words, the function of VHL protein (pVHL) was altered or even lost. As a part of the ubiquitin-mediated proteolysis pathway, the pVHL plays an important role in the degradation of several cellular proteins containing hypoxia-induced factors (HIF). HIF includes two subunits, namely, HIF1α and HIF2α, which participates in the transcription of some genes regulating metabolism and angiogenesis [17, 18]. Hence, the absence of pVHL function can result in the accumulation of HIF, which can contribute to the dysregulation of signaling pathways associated with metabolism, inflammation and angiogenesis, accelerating oncogenesis consequently [19]. Considering these published ideas together, we can speculate that the deleterious mutations in *VHL* identified in our study might play a leading role in the oncogenesis of ccRCC. However, loss of *VHL* activity is unable to induce ccRCC by itself, as there are some other ingredients cooperating with that towards the oncogenesis of ccRCC. Amrita and colleagues demonstrated that the deficiencies of *Vhl* and *Pbrm1* in the mouse kidney can lead to multifocal ccRCC with a tendency of metastasis [20]. Sabine and colleagues showed that the combined deletion of *Vhl*, *Trp53* and *Rb1* targeted in renal epithelial cells in mice caused ccRCC, which shared molecular markers and mRNA expression with human ccRCC [21].

As the second most frequently mutated gene in ccRCC both in the TCGA and COSMIC databases, *PBRM1* is located at chromosome 3p21 encoding the BAF180 protein, which is a vital component of the PBAF SWI/SNF chromatin remodeling complex [22]. In this study, only a stop-gain mutation (p.E981X) in *PBRM1* was detected in one ccRCC case, which had been reported previously. Compared with the data documented in the TCGA (30.6%) and COSMIC datasets (31%), the mutation frequency of *PBRM1* in ccRCC in this study was relatively lower (6.7%). Varela and partners disclosed truncating mutations in *PBRM1* at a frequency of 41% in 227 ccRCC cases [23]. The discrepancy in the mutation frequency of *PBRM1* in ccRCC between our study and previous studies might result from racial differences in the subjects. Moreover, the smaller sample amount in our study might also contribute to that, which should be taken into consideration. Therefore, additional analysis with a larger sample size still needs to confirm the data reported herein. Nowadays, it has been generally accepted that *PBRM1* acts as a tumor suppressor gene in the kidney and plays a critical role in the pathogenesis and progression of ccRCC [19]. It had been demonstrated that loss of *Vhl* and *Pbrm1* in mouse kidney could generate ccRCC [24]. As revealed in our study, *PBRM1* and *VHL* were somatically mutated in the same ccRCC case. Consequently, we speculated that somatically altered *PBRM1* and *VHL* genes worked cooperatively for the oncogenesis of ccRCC in our study. More recently, another study showed that depressed *PBRM1* and *VHL* expression was associated with elevated tumor aggressiveness [25]. In addition, the *PBRM1* mutation was also identified in one type 2 PRCC case in this study, which was consistent with the previous finding that mutated *PBRM1* was mainly associated with type 2 PRCC [4].

Apart from *VHL* and *PBRM1*, there are some other genes significantly mutated in ccRCC based on the TCGA and COSMIC datasets, such as *SETD2* and *BAP1*, which are both located at chromosome 3p21. For *BAP1*, a missense mutation (p.H193Q) and a frameshift-deletion (p.P352fs) were found in two different ccRCC cases in this study. Regarding *SETD2*, we also identified two somatic mutations in two distinct ccRCC cases consisting of a missense mutation (p.W1562C) and a stop-gain mutation (p.S512X). All of those mutations in *SETD2* and *BAP1* had not been reported before and were predicted to be deleterious. Serving as tumor suppressor genes in ccRCC, *BAP1* and *SETD2* mutations were related to worse cancer-specific survival [26]. In the TCGA database, only mutations in *BAP1* were reported to be associated with poor survival outcome [3]. Miura and colleagues unraveled in their research that deficiency of *BAP1* protein expression at metastatic sites indicated poor progression in patients with ccRCC [27]. Unfortunately, no prognostic information was available in our study. Thus, ccRCC patients who were confirmed to have *BAP1* and *SETD2* mutations should be followed up regularly. Further research with a larger sample size focusing on Chinese ccRCC patients, mainly concerning about the progression and prognosis of patients with altered *BAP1* and *SETD2*, should be considered.

It has been widely known that *TP53* is the most frequently mutated gene in ChRCC, with a frequency of 30.77% according to the TCGA dataset, which was only somatically mutated in one ChRCC case in this study and predicted to be deleterious. While Casuscelli and partners unraveled that *TP53* was mutated at a frequency of 58% in 38 metastatic ChRCC cases, which was much higher than that unmasked by the TCGA project and our study. In addition, those researchers found that mutations in *TP53* and *PTEN* and imbalanced chromosome duplication in primary ChRCC were associated with worse survival [28]. In contrast, all the specimens in our study were harvested from patients with no metastasis. It seemed that metastasis might underlie the discrepancy in the reported TP53 mutation frequencies. Thus, we hypothesised that somatically mutated *TP53* might serve as an important factor contributing to the aggressiveness of ChRCC. However, more further studies should be performed to confirm this hypothesis.

The PI3K/AKT/mTOR signaling pathway has been demonstrated to be highly involved in a variety of cancer types by contributing to the regulation of a series of cellular mechanisms, including proliferation, angiogenesis, metastasis and survival [29]. It was also reported that the PI3K/AKT/mTOR signaling pathway was significantly altered and activated in ccRCC [3, 14], playing a dominant role in the tumorigenesis in distal tubules of rats and human beings [30]. In our study, a multitude of somatically mutated genes associated with the PI3K/AKT signaling pathway were identified in all the three RCC types, while none of the mutated genes was involved in the mTOR signaling pathway. As an important therapeutical target, mTOR inhibitors, such as everolimus, have been recommended for the treatment of patients with metastatic ccRCC. To the best of our knowledge, investigations concerning about mTOR inhibitors and metastatic RCC have been launched to search for predictive factors among the components of the PI3K/AKT/mTOR signaling pathway [31]. However, in order to better use mTOR inhibitors for the treatment of metastatic RCC, further more studies focusing on the correlation between the PI3K/AKT/mTOR signaling pathway and RCC are still required.

Currently, PD-L1 expression in tumor cells has become a predictor of the response to immunotherapy with PD-1/PD-L1 inhibitors among diverse cancers including RCC [6]. In this study, among the 26 RCC cases, PD-L1-positive rate in tumor cells was significantly higher in specimens with 4 somatically mutated genes, including *CSPG4*, *DNAH11*, *INADL* and *TMPRSS13*, than in samples without those (P < 0.05). None of those gene mutations were reported to correlate with PD-L1 expression in RCC tumor cells previously. In other words, those altered genes could serve as predictors of the PD-L1-positive expression in RCC tumor cells. Consequently, it could be speculated that those four somatically mutated genes might become the potential targeted genes for predicting responses to immunotherapy with PD-1/PD-L1 inhibitors in RCC. Nevertheless, whether those four mutated genes can influence the expression of PD-L1 in RCC is in need of further investigation. Previous studies had revealed that PD-L1 expression had an association with poor overall survival in ccRCC [32], while the TCGA database indicated that only mutations in *BAP1* were associated with poor survival in ccRCC [3]. Both of somatically mutated *BAP1* and PD-L1 expression were demonstrated to correlate with the poor prognosis of ccRCC patients. As revealed in this study, *BAP1* was altered in only two ccRCC specimens, both of which exhibited PD-L1-positive in tumour cells. Those two mutations in *BAP1* were predicted to be deleterious. Therefore, we hypothesised that somatically altered *BAP1* might serve as a critical ingredient contributing to the PD-L1 expression in ccRCC tumor cells, and most likely work in concert with PD-L1 in tumor cells contributing to the aggressiveness of ccRCC. The interaction between somatic mutations in *BAP1* and PD-L1 expression in ccRCC needs to be further elucidated in additional studies.

## Conclusion

We identified somatic mutations in RCC from 26 Chinese patients using WES, which enriched the racial diversity of the somatic mutation profiles of RCC subjects. Several discrepancies in molecular characterizations were elucidated, such as the significant difference in the most frequent substitution in somatic missense mutations between our study and published databases. We also detected numerous novel somatic mutations in this study, which further supplements the somatic mutation profiles of RCC. Moreover, our study revealed that 4 somatically mutated genes, including *CSPG4*, *DNAH11*, *INADL* and *TMPRSS13*, might act as promising predictive factors of PD-L1-positive expression in RCC tumor cells.

## Additional file

**Additional file 1: Table S1.** Additional table.

## Abbreviations

RCC: renal cell carcinoma
ccRCC: clear cell renal cell carcinoma
PRCC: papillary renal cell carcinoma
ChRCC: chromophobe renal cell carcinoma
NGS: next generation sequencing
WES: whole exome sequencing
TCGA: The Cancer Genome Atlas
ICGC: International Cancer Genome Consortium
TMB: tumor mutation burden
gDNA: genomic DNA
BWA: Burrows Wheeler Aligner
GATK: Genome Analysis Toolkit
ACMG: American College of Medical Genetics
KEGG: Kyoto Encyclopedia of Genes and Genomes
pVHL: VHL protein
HIF: hypoxia-induced factor
PD-L1: programmed death ligand 1
PD-1: programmed death 1
